# Interleukin-16 Promotes Cardiac Fibrosis and Myocardial Stiffening in Heart Failure with Preserved Ejection Fraction

**DOI:** 10.1371/journal.pone.0068893

**Published:** 2013-07-19

**Authors:** Shunsuke Tamaki, Toshiaki Mano, Yasushi Sakata, Tomohito Ohtani, Yasuharu Takeda, Daisuke Kamimura, Yosuke Omori, Yasumasa Tsukamoto, Yukitoshi Ikeya, Mari Kawai, Atsushi Kumanogoh, Keisuke Hagihara, Ryohei Ishii, Mitsuru Higashimori, Makoto Kaneko, Hidetoshi Hasuwa, Takeshi Miwa, Kazuhiro Yamamoto, Issei Komuro

**Affiliations:** 1 Department of Cardiovascular Medicine, Osaka University Graduate School of Medicine, Suita, Japan; 2 Genome Information Research Center, Osaka University, Suita, Japan; 3 Department of Medical Science and Cardiorenal Medicine, Yokohama City University Graduate School of Medicine, Yokohama, Japan; 4 Division of Cardiology, Department of Internal Medicine, Nihon University School of Medicine, Tokyo, Japan; 5 Department of Respiratory Medicine, Allergy and Rheumatic Diseases, Osaka University Graduate School of Medicine, Suita, Japan; 6 Department of Kampo Medicine, Osaka University Graduate School of Medicine, Suita, Japan; 7 Department of Mechanical Engineering, Osaka University, Suita, Japan; 8 Department of Molecular Medicine and Therapeutics, Faculty of Medicine, Tottori University, Yonago, Japan; Temple University, United States of America

## Abstract

**Background:**

Chronic heart failure (CHF) with preserved left ventricular (LV) ejection fraction (HFpEF) is observed in half of all patients with CHF and carries the same poor prognosis as CHF with reduced LV ejection fraction (HFrEF). In contrast to HFrEF, there is no established therapy for HFpEF. Chronic inflammation contributes to cardiac fibrosis, a crucial factor in HFpEF; however, inflammatory mechanisms and mediators involved in the development of HFpEF remain unclear. Therefore, we sought to identify novel inflammatory mediators involved in this process.

**Methods and Results:**

An analysis by multiplex-bead array assay revealed that serum interleukin-16 (IL-16) levels were specifically elevated in patients with HFpEF compared with HFrEF and controls. This was confirmed by enzyme-linked immunosorbent assay in HFpEF patients and controls, and serum IL-16 levels showed a significant association with indices of LV diastolic dysfunction. Serum IL-16 levels were also elevated in a rat model of HFpEF and positively correlated with LV end-diastolic pressure, lung weight and LV myocardial stiffness constant. The cardiac expression of IL-16 was upregulated in the HFpEF rat model. Enhanced cardiac expression of IL-16 in transgenic mice induced cardiac fibrosis and LV myocardial stiffening accompanied by increased macrophage infiltration. Treatment with anti-IL-16 neutralizing antibody ameliorated cardiac fibrosis in the mouse model of angiotensin II-induced hypertension.

**Conclusion:**

Our data indicate that IL-16 is a mediator of LV myocardial fibrosis and stiffening in HFpEF, and that the blockade of IL-16 could be a possible therapeutic option for HFpEF.

## Introduction

Despite the progress in pharmacologic therapies, chronic heart failure (CHF) remains a major public health problem [1]. Approximately half of all patients with CHF have a preserved left ventricular (LV) ejection fraction, commonly referred to as heart failure with preserved ejection fraction (HFpEF) [2], [3]. Therapies with proven benefit in heart failure with reduced ejection fraction (HFrEF) have failed to improve outcomes in HFpEF patients [3], [4], which strongly suggests a different pathophysiology between HFpEF and HFrEF and the need for identification of a specific therapeutic target for HFpEF.

The primary cause of HFpEF has been attributed to an abnormality in diastolic function of the left ventricle, although the involvement of other factors such as increased arterial stiffness, sodium retention or neurohormonal activation in the development of HFpEF has also been suggested [5]. LV diastolic function has been divided into active relaxation and LV passive stiffness, and an abnormal elevation in LV passive stiffness has been shown in HFpEF patients [5]. Using an animal model of HFpEF, we clarified that LV stiffening plays a crucial role in the transition from asymptomatic diastolic dysfunction to HFpEF, and that LV myocardial fibrosis is an important cause of LV stiffening [6], [7].

Recent evidence has shown that activation of the immune system plays an important role in CHF. Immune activation caused by myocardial injury, bacterial translocation and peripheral tissue hypoxia is thought to result in the production of pro-inflammatory mediators including tumor necrosis factor-α, interleukin (IL)-1β and IL-6 from mononuclear cells or the myocardium itself. These mediators have been reported to worsen CHF through their detrimental effect on myocardial contractility, LV remodeling or endothelial function [8], [9]. Increased circulating levels of cytokines or chemokines have been shown to be associated with the severity of clinical symptoms and increased mortality [10], [11]. However, these have been reported mainly in HFrEF patients or experimental models of CHF. There have been a few studies showing the association between cardiac inflammation and cardiac fibrosis or diastolic dysfunction [12]–[14], but the role of the immune system and specific inflammatory mediators involved in the development of HFpEF is not clear. Chronic inflammatory reactions promote fibrotic tissue remodeling, which can affect all organ systems including the heart [15], [16]. From our previous studies and other studies, cardiac inflammation seems to be associated with the fibrotic process in HFpEF [17], [18].

In this study, we aimed to identify novel inflammatory mediators associated with the development of HFpEF. Our results suggested that IL-16, a cytokine which has been shown to be a key mediator of several inflammatory, allergic, or infectious diseases [19]–[21], promotes myocardial fibrosis, leading to increased LV myocardial stiffness.

## Methods

The clinical study was approved by Osaka University Hospital Ethical Committee (Permit Number: 09056-2, 10081-3), and conducted in accordance with the Declaration of Helsinki. All participants gave written informed consent. The experimental study was approved by the institutional ethics committee of Osaka University Graduate School of Medicine (Permit Number: 23-014-0, 23-030-1, 23-062-0), and conformed to the Guide for the Care and Use of Laboratory Animals published by the United States National Institutes of Health.

### Study Patients

Blood samples and echocardiograms were obtained from patients in Osaka University Hospital with a history of hospital admission for heart failure. Heart failure was clinically diagnosed according to the criteria used in the Framingham Heart Study project [22]. Patients with LV ejection fraction >40% and those with ≦ 40% were defined as HFpEF and HFrEF, respectively [4]. All patients were required to be in the compensated state at the time of blood sampling and echocardiography. Patients were excluded from this study if they had acute coronary syndrome, ischemic cardiomyopathy, congenital heart disease, severe valvular disease, myocarditis, epicarditis, amyloidosis, significant renal dysfunction (serum creatinine level >2.0 mg/dl), active infectious diseases or cancer. Patients were also excluded if they had a history of bronchial asthma or any allergic, inflammatory or granulomatous disease, or were receiving systemic or topical corticosteroid therapy or any other immunomodulating medications. Medications were not withheld in the patients with HFpEF or HFrEF for ethical reasons. Healthy volunteers served as a control group. Venous blood was drawn from a superficial forearm vein following an overnight fast. Serum was obtained by allowing the blood sample to clot at room temperature for 1 hour followed by centrifugation.

### Echocardiography in Human Subjects

Transthoracic echocardiography was performed according to standard techniques using a commercially available machine as previously described [23], [24]. LV ejection fraction was calculated by Teichholz’s formula or Simpson’s rule. The LV mass index and relative wall thickness were calculated, early transmitral flow velocity (*E*) was measured by pulsed-wave Doppler, and the septal mitral annular early diastolic velocity (e’) was determined by spectral tissue Doppler imaging using standard methods as previously described [23]. Left atrial volume index (LAVI) was calculated in the apical 4-chamber view by the single-plane, area-length method [25]. Diastolic wall strain (DWS), a non-invasive index of LV passive stiffness, was calculated as follows: DWS = (PWs – PWd)/PWs, where PWs indicates posterior wall thickness at end-systole and PWd indicates posterior wall thickness at end-diastole [23], [26]. Systolic and diastolic blood pressure and heart rate were measured at the time of echocardiography.

### Multiplex-bead Array Assay

Human serum samples were analyzed using Bio-Plex human cytokine 23-plex and 27-plex panel assays (Bio-Rad). The assay was performed according to the manufacturer’s protocol. The resulting raw data were collected using the Bio-Plex 200 system (Bio-Rad) and analyzed using Bio-Plex Manager 5.0 software (Bio-Rad).

### HFpEF Rat Model

Male Dahl salt-sensitive rats (SLC Japan) were fed a high-salt (8% NaCl) diet (Oriental Yeast Co.) starting at 6 weeks of age and served as the hypertensive HFpEF model as previously described [6], [7], [17], [23], [27]. Male Dahl salt-sensitive rats fed 0.3% NaCl chow served as age-matched controls. The data were obtained around 21 weeks of age, when this HFpEF model shows signs of overt heart failure with increased LV filling pressure and pulmonary congestion, without any significant changes in LV dimensions or fractional shortening [6], [7], [17], [23], [27]. Systolic blood pressure was measured with a tail-cuff system (BP-98A, Softron).

### Echocardiography, Hemodynamic Studies and Tissue Sampling in Rats

Rats were anesthetized with intraperitoneal ketamine and xylazine (80 and 10 mg/kg, respectively), transthoracic echocardiography was performed and M-mode echocardiograms were recorded using an echocardiographic machine equipped with a 12-MHz transducer (SONOS 5500, Philips Medical System), as previously described [27]. The adequacy of anesthesia was monitored by the stability of blood pressure, heart rate and lack of flexor responses to a paw-pinch. A 1.5-F, high-fidelity, manometer-tipped catheter (SPR-407, Millar Instruments) was introduced through the right carotid artery into the left ventricle to determine the LV end-diastolic pressure (LVEDP), the time constant of LV relaxation (Tau), and the myocardial stiffness constant (MSC) as previously described [27]. Following the hemodynamic study and additional anesthesia, blood was collected from the *vena cava*, and rats were euthanized by removal of the heart. The heart and lungs were rapidly harvested and weighed.

### Transgenic Mice

Mouse IL-16 cDNA was isolated by reverse transcriptase-polymerase chain reaction from mouse spleen total RNA and cloned into the plasmid containing the α-myosin heavy chain (α-MHC) promoter and a simian virus 40 polyadenylation site. The construct was linearized, gel purified and microinjected into the pronuclei of BDF1 (C57BL/6 × DBA/2) mouse zygotes. Transgenic (TG) mice were identified by PCR, with primers specific for the α-MHC promoter and IL-16 cDNA. IL-16 TG mice were crossed with C57BL/6 mice (SLC Japan), and the male F2 mice were used in the present study. The littermates of TG mice were used as non-transgenic (Non-TG) mice. All mice were analyzed at 20–22 weeks of age.

### A Mouse Model of Hypertension and IL-16 Neutralization

ALZET osmotic minipumps (DURECT Corp.) were implanted subcutaneously in male C57BL/6 mice (SLC Japan) at 8–10 weeks of age for the administration of angiotensin II (Ang II) (1.2 mg/kg/day; A-9525, Sigma-Aldrich, Inc.) for 14 or 28 days. Osmotic minipumps containing saline were implanted in control mice. Mice were anesthetized with intraperitoneal ketamine and xylazine (100 and 10 mg/kg, respectively) to implant the osmotic minipumps. The adequacy of anesthesia was determined by the absence of a pedal reflex. To block the effect of IL-16, a group of Ang II-treated mice received an intraperitoneal injection of 200 µg anti-IL-16 neutralizing monoclonal antibody clone 14.1 (BD Biosciences) 3 times per week starting 1 day before and continuing until 14 or 28 days after the implantation of the osmotic minipumps, based on previous reports [28], [29]. The other groups of Ang II-treated mice and the saline-infused control mice received an intraperitoneal injection of phosphate buffered saline (PBS).

### Echocardiography and Tissue Sampling in Mice

Transthoracic echocardiography was performed in conscious mice using the Vevo 770 Imaging System equipped with a 25-MHz linear probe (Visual Sonics). After echocardiography, mice were adequately anesthetized with intraperitoneal ketamine and xylazine (100 and 10 mg/kg, respectively) and euthanized by removal of the heart. An adequate anesthetic depth was determined by the absence of the pedal reflex. The heart and lungs were quickly harvested, and hearts were then promptly perfused through the aorta with ice-cold Ca^2+^-free Tyrode’s solution containing 30 mM 2,3-butanedione monoxime (BDM). The left ventricle was sectioned perpendicularly to the longitudinal axis to obtain a transverse section at the mid-level of the heart with a 2–3 mm thickness, and this was used for the measurement of myocardial stiffness. LV samples for immunohistochemistry were embedded in Tissue Tek OCT compound (Sakura Finetechnical Co. Ltd.). The apical part of LV myocardium was snap-frozen in liquid nitrogen and stored for the measurement of mRNA and protein levels. The rest of the LV specimen was fixed with phosphate-buffered 10% formalin solution, embedded in paraffin, and 3 µm thick transverse cross-sections from the midventricular plane were stained with Sirius Red.

### Measurement of LV Myocardial Stiffness in Mice

Skinned LV muscles from mice were prepared according to previously reported methods [30]. Transverse sections of the left ventricle were skinned in relaxing solution (5 mM MgATP, 40 mM BES, 1 mM Mg^2+^, 10 mM EGTA, 1 mM dithiothreitol, 15 mM phosphocreatine, 15 U/ml creatine phosphokinase, 10 mM BDM, 180 mM ionic strength [adjusted by K-propionate], pH 7.0) containing 1% Triton X-100 overnight. The specimens were then washed thoroughly with relaxing solution and stored in relaxing solution containing 50% glycerol. All solutions contained protease inhibitors (0.5 mM PMSF, 0.04 mM leupeptin and 0.01 mM E64).

We used a balloon-type sensing system to evaluate LV myocardial stiffness [31]. The skinned transverse section was placed around a latex balloon (Labo Support), while the pressure inside the balloon was monitored. The balloon was then dilated with the deformation information of the balloon and the specimen captured by a CCD camera. Young’s modulus *E_H_* was obtained from the internal pressure of the balloon and the strain of the transverse LV section based on a dual cylinder model.

### Western Blot Analysis

Proteins were extracted from the left ventricle of the mice as previously described [32]. Proteins were separated on SDS-PAGE gels and transferred to PVDF membranes (Millipore). Membranes were probed with antibodies to IL-16 (1∶200; MAB1727, R&D Systems), Collagen I (1∶500; AB765P, Millipore), transforming growth factor-beta 1 (TGF-β1) (1∶200; sc-146, Santa Cruz Biotechnology, Inc.) and connective tissue growth factor (CTGF) (1∶5000; ab6992, Abcam). Blots were developed using enhanced chemiluminescence and expression levels were quantified using LAS-4000 and MultiGauge software (Fujifilm). The band density of the protein of interest was normalized to GAPDH expression (1∶10000; sc-25778, Santa Cruz Biotechnology, Inc.).

### Immunohistochemistry

Cryostat-frozen LV transverse cross-sections from the midventricular plane (8µm thick) were labeled with anti-IL-16 (1∶100; sc-7902, Santa Cruz Biotechnology, Inc.), anti-F4/80 (1∶100; MCA497, Serotec) and anti-TGF-β1 (1∶50; sc-146-G, Santa Cruz Biotechnology, Inc.) antibodies. Fluorophore-conjugated secondary antibodies (Invitrogen) were applied, and stained samples were mounted with ProLong Gold antifade reagent with DAPI (Invitrogen). To evaluate macrophage infiltration, images of 12 random regions of the section were captured at ×400 magnification using a fluorescence microscope (BZ-9000, Keyence), and F4/80-positive cells were counted and expressed as cells per square millimeter of myocardium as previously described [33]. Confocal images were obtained using a laser scanning microscope (TCS SP5, Leica).

### Measurement of Myocardial Fibrosis

National Institutes of Health ImageJ software (Version 1.45) was used to measure the amount of myocardial fibrosis on sections stained by Sirius Red. In each section, 5 fields were randomly selected and the percent area of fibrosis was determined by the ratio of the Sirius Red-stained area to total myocardial area [34]. Fibrosis of the perivascular, epicardial and endocardial areas were excluded from the measurements [35].

### Cell Culture

Peritoneal cells were collected from male C57BL/6 mice (SLC Japan) at 6–8 weeks of age by peritoneal lavage with 10 ml of PBS after euthanasia by cervical dislocation. The cells were centrifuged, resuspended in DMEM supplemented with 10% FBS and incubated on a 12-well plate (2×10^6^ cells/well). The cells were incubated to allow macrophages to adhere to the bottom of the plates. The plates were then washed gently with PBS to remove nonadherent cells, and the macrophages were incubated in serum-free medium containing murine recombinant IL-16 (Shenandoah Biotechnology Inc.) for 24 h. Supernatants were collected from the cell cultures.

### Enzyme-linked Immunosorbent Assay

The concentration of IL-16 in human, rat and mouse serum samples was measured by commercially available enzyme-linked immunosorbent assay (ELISA) kits specific for human (R&D Systems), rat and mouse (Cusabio) IL-16, respectively. The concentration of TGF-β1 in the macrophage culture medium was determined using a commercially available ELISA kit for mouse TGF-β1 (R&D Systems).

### Quantification of Gene Expression

Total RNA was isolated from the left ventricle, and the mRNA level was quantified by real-time quantitative polymerase chain reaction with the ABI PRISM 7900 HT Sequence Detection System and Software (Applied Biosystems) as previously described [32]. Sequences of primers and probes and TaqMan gene expression assay IDs purchased from Applied Biosystems were as follows: rat IL-16: Rn01477714_g1; rat GAPDH: Rn99999916_s1; mouse Collagen I: Mm00801666_g1; mouse TGF-β1: forward 5′-TGA CGT CAC TGG AGT TGT ACG G-3′, reverse 5′-GGT TCA TGT CAT GGA TGG TGC-3′, TaqMan probe 5′-TTC AGC GCT CAC TGC TCT TGT GAC AG-3′; mouse CTGF: forward 3′-AGC CGC CTC TGC ATG GTC A-3′, reverse 5′-GCG ATT TTA GGT GTC CGG AT-3′, TaqMan probe, 5′-CCT GCG AAG CTG ACC TGG AGG AAA-3′; mouse IL-16: Mm01317937_g1; mouse F4/80: Mm00802529_m1; mouse GAPDH: Mm99999915_g1. All data were normalized to GAPDH expression.

### Statistical Analysis

Data are presented as mean ± SEM. Data were analyzed using statistical software (StatView version 5.0, SAS Institute Inc.). Differences between two groups for continuous and discrete variables were analyzed with an unpaired Student’s *t*-test and Fisher’s exact test, respectively. Differences among more than two groups were assessed by one-factor ANOVA followed by a Tukey-Kramer multiple comparison test. Correlations between variables were determined by Pearson’s correlation coefficient. A *P* value <0.05 was considered statistically significant.

## Results

### Serum IL-16 Levels are Elevated in HFpEF Patients and Associated with LV Diastolic Dysfunction

**Figure 1:**
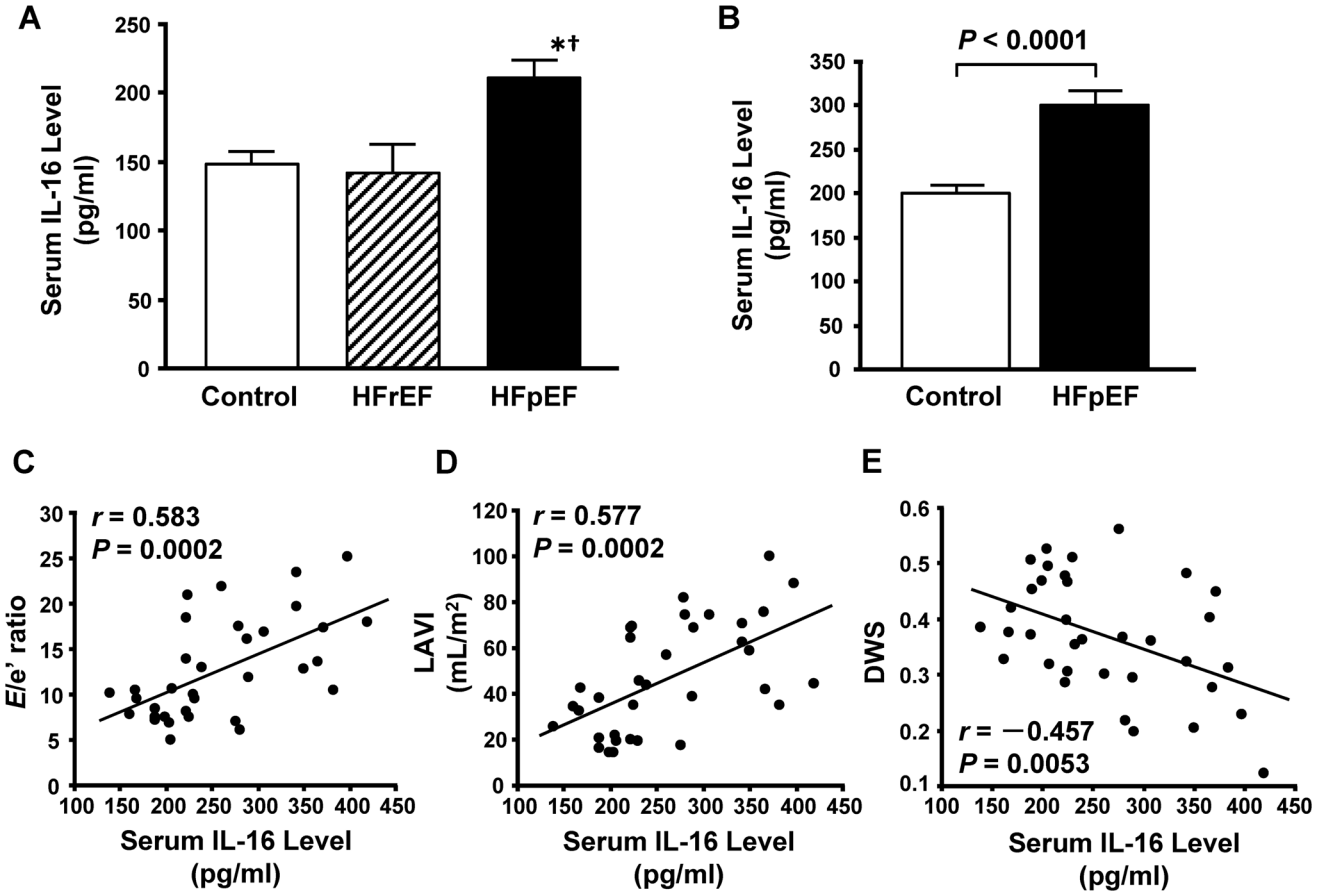
Elevated serum interleukin-16 (IL-16) levels in patients with heart failure with preserved ejection fraction. **A**, Serum IL-16 levels in controls and patients with heart failure with reduced (HFrEF) or preserved ejection fraction (HFpEF) measured by a multiplex-bead array assay. *P<0.05 vs. control group. ^†^ *P*<0.05 vs. HFrEF group. **B**, Serum IL-16 levels in controls and patients with HFpEF measured by enzyme-linked immunosorbent assay. **C** through **E**, Correlations of serum IL-16 level and the ratio of early transmitral flow velocity to septal mitral annular early diastolic velocity (*E*/e′ ratio) (**C**), left atrial volume index (LAVI) (**D**) and diastolic wall strain (DWS) (**E**) in controls and HFpEF patients combined.

First, we analyzed serum samples from the HFpEF and HFrEF patients and controls (*Table 1*) using a multiplex-bead array assay for screening the 50 cytokines, chemokines, growth factors, angiogenic factors and soluble receptors. This analysis revealed that serum IL-16 levels were significantly higher in patients with HFpEF than in patients with HFrEF or in controls (*Figure 1A*). Although we also found significant differences in several analytes other than IL-16 among the three groups (*Table 2*), we decided to focus on IL-16 because of the specific increase of IL-16 in HFpEF patients.

**Table 1:** Clinical and study characteristics of controls and the patients with heart failure with reduced or preserved ejection fraction.

	Control(*n* = 8)	HFrEF(*n* = 9)	HFpEF(*n* = 11)
Age, y	65±1	56±5	78±2* †
NYHA class I/II/III/IV	–	1/4/3/1	0/3/8/0
Body mass index, kg/m^2^	22.9±1.0	23.2±0.7	24.6±1.5
BNP, pg/ml	21±5	106±46	129±27
**Echocardiography**			
LV end-diastolic dimension, mm	43.4±1.3	64.7±1.5*	46.4±1.8†
EF, %	64.8±1.9	31.1±1.9*	67.9±2.3†

Data are mean ± SEM. HFrEF and HFpEF indicate heart failure with reduced and preserved ejection fraction, respectively; NYHA, New York Heart Association; BNP, brain natriuretic peptide; LV, left ventricular; and EF, ejection fraction.

* *P*<0.05 vs. control group,

† *P*<0.05 vs. HFrEF group.

**Table 2:** Serum levels of analytes (excluding IL-16) that were significantly different among the controls and two patient groups.

	Control(*n* = 8)	HFrEF(*n* = 9)	HFpEF(*n* = 11)
MIG	692.0±50.5	1525.4±533.1	5470.8±1594.6* †
SCF	106.4±6.0	131.7±17.3	206.2±21.4* †
Eotaxin	43.3±5.2	43.5±5.3	70.0±9.4* †
IP-10	413.5±60.8	564.1±68.9	853.1±111.0*

Data are mean ± SEM. Values are in pg/ml. HFrEF and HFpEF indicate heart failure with reduced and preserved ejection fraction, respectively; MIG, monokine induced by interferon-γ; SCF, stem cell factor; and IP-10, interferon-inducible protein 10.

* *P*<0.05 vs. control group,

† *P*<0.05 vs. HFrEF group.

Next, we included more HFpEF patients and controls, and measured the serum IL-16 levels by ELISA to confirm the results obtained by the multiplex-bead array assay. The characteristics of the total study patients are shown in *Table 3* *.* LV ejection fraction was not significantly different between the two groups, whereas LV end-systolic and diastolic dimensions were significantly larger in the HFpEF group than in the control group, which is consistent with previous reports [36]. Analysis of serum levels of IL-16 in this larger population confirmed that IL-16 was significantly higher in HFpEF patients than in controls (*Figure 1B*). Moreover, serum levels of IL-16 were positively correlated with the *E*/e′ ratio and LAVI when both groups were combined (*Figure 1C and D*). These results suggested a possible association between IL-16 and indices of LV diastolic dysfunction and/or elevation of LV filling pressure in human subjects. In addition, we observed a correlation between serum IL-16 levels and DWS, suggesting that elevation of IL-16 is associated with LV stiffening in human subjects (*Figure 1E*).

**Table 3:** Clinical and study characteristics of controls and the patients with heart failure with preserved ejection fraction.

	Control (*n* = 14)	HFpEF (*n* = 21)
Age, y	61±2	70±3
Male sex, *n* (%)	5 (36)	11 (52)
NYHA class I/II/III/IV	–	3/9/9/0
Height, m	1.58±0.02	1.59±0.02
Body weight, kg	55±2	62±3*
Body mass index, kg/m^2^	22.1±0.6	24.6±1.1
Systolic blood pressure, mmHg	124±4	124±4
Diastolic blood pressure, mmHg	75±2	66±3*
Heart rate, bpm	67±4	67±3
Hemoglobin, g/dL	13.8±0.3	12.5±0.4*
Creatinine, mg/dL	0.63±0.05	1.23±0.06*
eGFR, mL/min/1.73m^2^	86±3	44±4*
BNP, pg/ml	17±3	216±57*
**Echocardiography**		
LV end-diastolic dimension, mm	44.4±0.8	48.3±1.3*
LV end-systolic dimension, mm	27.9±0.9	32.2±1.3*
IVSd, mm	7.0±0.3	11.0±0.6*
PWd, mm	7.0±0.2	9.5±0.5*
LV mass index, g/m^2^	61.3±3.1	112.1±8.8*
RWT	0.32±0.01	0.40±0.02*
EF, %	67.3±1.6	61.4±2.3
*E*, m/s	0.64±0.05	0.82±0.06*
DcT, ms	191±15	208±13
e′, cm/s	7.6±0.6	5.5±0.4*
*E*/e′ ratio	8.7±0.6	15.5±1.2*
LAVI, mL/m^2^	24.7±2.8	61.3±4.1*
DWS	0.45±0.02	0.32±0.02*

Data are mean ± SEM. HFpEF indicates heart failure with preserved ejection fraction; NYHA, New York Heart Association; eGFR, estimated glomerular filtration rate; BNP, brain natriuretic peptide; LV, left ventricular; IVSd, interventricular wall thickness at end-diastole; PWd, LV posterior wall thickness at end-diastole; RWT, relative wall thickness; EF, ejection fraction; *E*, early transmitral flow velocity; DcT, deceleration time of early transmitral flow velocity; e′, septal mitral annular early diastolic velocity; LAVI, left atrial volume index; and DWS, diastolic wall strain.

* *P*<0.05 vs. control group.

### Serum IL-16 Levels and Cardiac Expression of IL-16 are Elevated in the HFpEF Rats

To examine whether the elevation in serum IL-16 and its association with diastolic dysfunction is a common phenomenon in HFpEF, we analyzed a rat model of HFpEF. The changes in myocardial anatomy and function induced by hypertension in our HFpEF model (*Table 4*) were similar to changes that have been described in previous reports [6], [7], [17], [23], [27].

**Table 4:** Hemodynamic, pathological and echocardiographic parameters of Dahl salt-sensitive rats.

	Control (*n* = 8)	HFpEF (*n* = 12)
Body weight, g	422±10	389±7*
Systolic blood pressure, mmHg	129±3	219±5*
Heart rate, bpm	393±12	452±9*
LV weight/body weight, mg/g	2.09±0.02	3.28±0.10*
Lung weight/body weight, mg/g	3.44±0.08	4.39±0.23*
**Echocardiography**		
LV end-diastolic dimension, mm	9.47±0.15	9.09±0.18
PWd, mm	1.10±0.02	1.73±0.04*
Fractional shortening, %	31±1	33±1
Midwall fractional shortening, %	19±1	19±1
**Catheterization**		
LV end-diastolic pressure, mmHg	1.8±0.4	7.3±1.2*
Tau, ms	16±1	28±3*
MSC	2.4±0.2	6.3±0.4*

Data are mean ± SEM. HFpEF indicates heart failure with preserved ejection fraction; LV, left ventricular; PWd, LV posterior wall thickness at end-diastole; Tau, time constant of LV relaxation; and MSC, myocardial stiffness constant.

* *P*<0.05 vs. control group.

**Figure 2:**
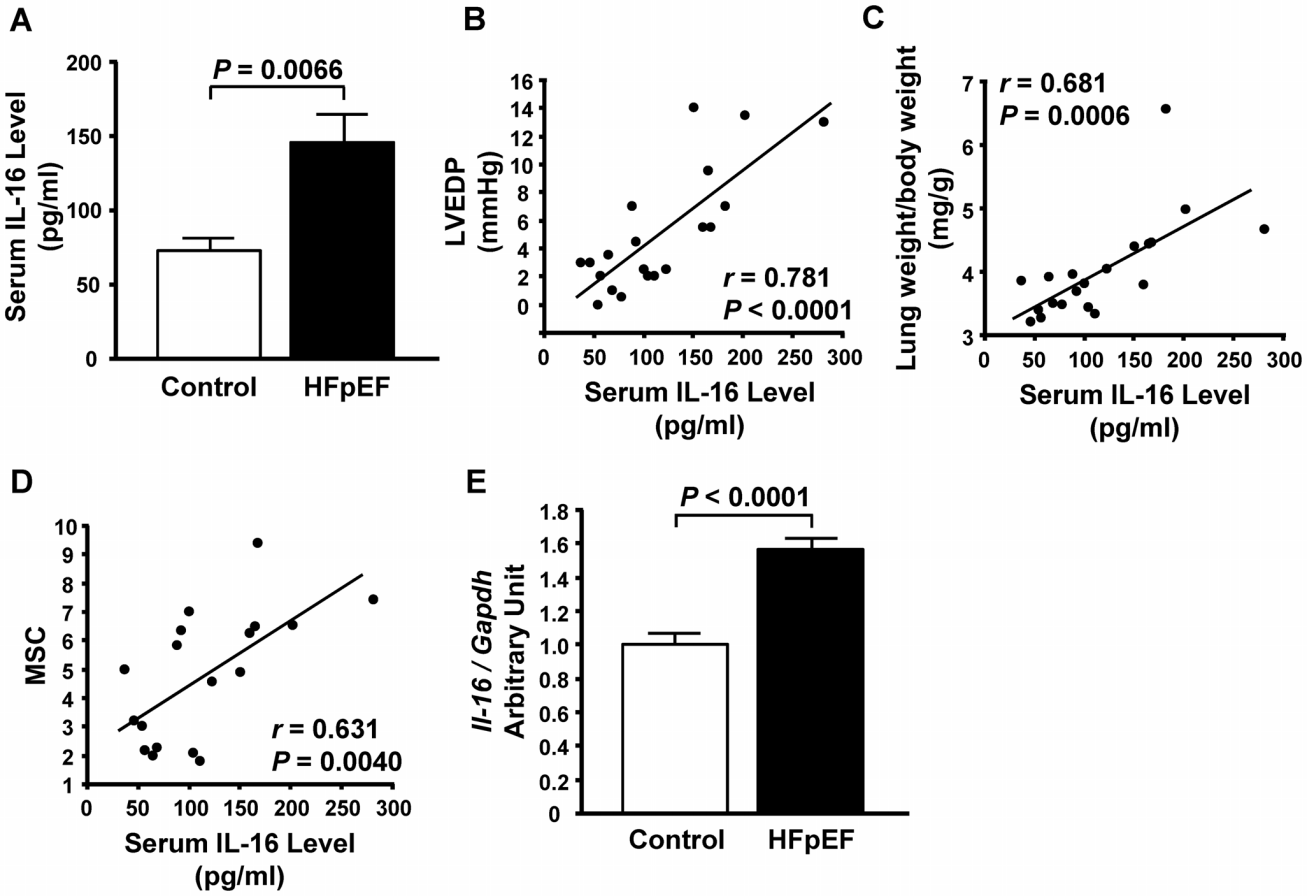
Elevated serum interleukin-16 (IL-16) levels in rats with heart failure with preserved ejection fraction (HFpEF). **A,** Serum IL-16 levels in control rats and the rats with HFpEF. **B** through **D,** Correlations of serum IL-16 level and left ventricular end-diastolic pressure (LVEDP) (**B**), lung weight to body weight ratio (lung weight/body weight) (**C**) and myocardial stiffness constant (MSC) (**D**) in control and HFpEF rats combined. **E,** mRNA level of IL-16 in the left ventricle of control and HFpEF rats.

The serum levels of IL-16 were significantly higher in rats with HFpEF than in control rats (*Figure 2A*), and positively correlated with LVEDP and the ratio of lung weight to body weight in all rats (*Figure 2B and C*). Although there was no correlation between serum IL-16 levels and Tau (*r* = 0.331, *P* = 0.1561), we found a positive correlation between serum IL-16 levels and MSC (*Figure 2D*), suggesting that elevation of IL-16 in HFpEF rats is associated with LV myocardial stiffening but not with LV abnormal relaxation. Furthermore, expression of IL-16 mRNA in the left ventricle was higher in HFpEF rats than in control rats (*Figure 2E*).

### Enhanced Cardiac Expression of IL-16 causes Myocardial Fibrosis and Stiffness

To assess the effect of enhanced cardiac expression of IL-16, we generated TG mice carrying murine IL-16 cDNA under the control of the α-MHC promoter. We identified four transgene positive founders by PCR. Among them, germline transmission was observed in three lines (line 13, 21, and 22). The line 21 mice expressing the highest myocardial levels of the transgene were bred and analyzed.

**Figure 3:**
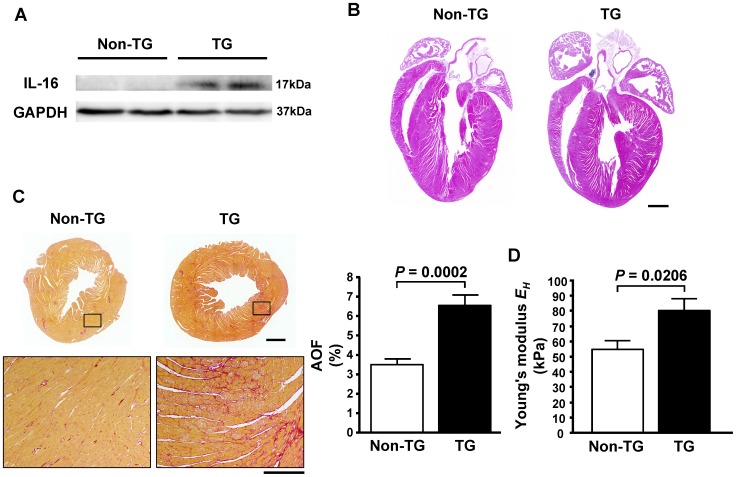
Enhanced cardiac expression of interleukin-16 (IL-16) causes increased myocardial fibrosis and stiffness in mice. **A**, Cardiac expression of IL-16 protein in non-transgenic (Non-TG) and transgenic (TG) mice. **B**, Four-chamber view of the hearts from Non-TG and TG mice stained with hematoxylin and eosin. Bar = 1 mm. **C**, Representative photomicrograph of Sirius Red-stained heart sections and the percent area of fibrosis (AOF) of Non-TG and TG mice. Bar: Upper panel = 1 mm; Lower panel = 200 µm. **D**, Young’s modulus *E_H_* in Non-TG and TG mice.

**Figure 4:**
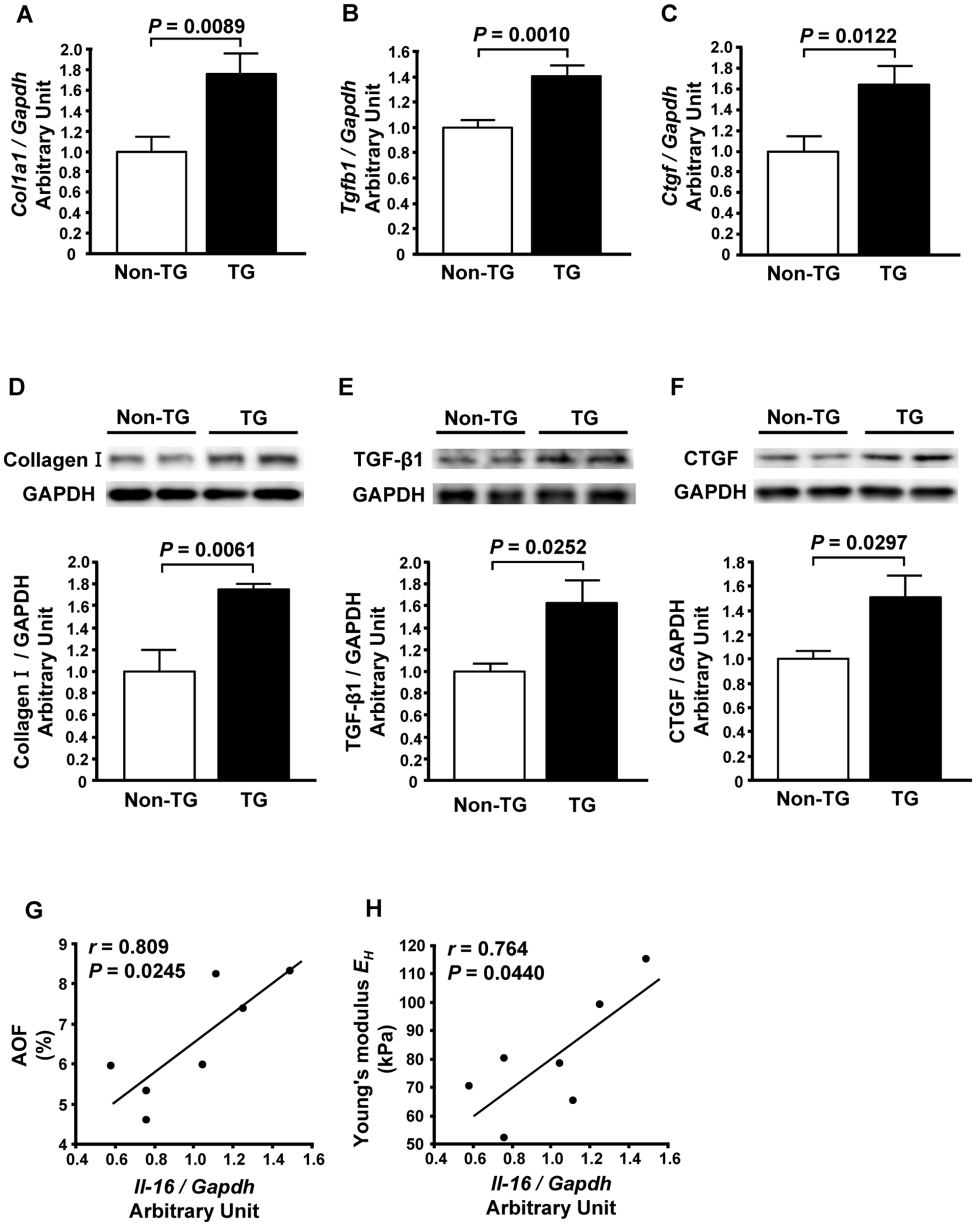
Effect of enhanced cardiac expression of interleukin-16 (IL-16) on markers of cardiac fibrosis in mice. **A** through **C**, Left ventricular mRNA levels of Collagen I (**A**), transforming growth factor-beta 1 (TGF-β1) (**B**) and connective tissue growth factor (CTGF) (**C**) in non-transgenic (Non-TG) and transgenic (TG) mice. **D** through **F**, Left ventricular protein levels of Collagen I (**D**), TGF-β1 (**E**) and CTGF (**F**) in Non-TG and TG mice. Top panels in each figure show a representative Western blot. *n* = 5 per group. **G** and **H**, Correlations of IL-16 mRNA levels with AOF (**G**) and Young’s modulus *E_H_* (**H**) in TG mice.

The enhanced expression of the bioactive secreted form of IL-16 in the heart of TG mice was confirmed by Western blotting (*Figure 3A*), whereas there was no significant difference in serum IL-16 levels between TG (32.1±6.8 pg/ml) and Non-TG (31.3±6.0 pg/ml) mice. Atrial enlargement was observed (*Table 5* and *Figure 3B*) and the extent of LV fibrosis was increased (*Figure 3C*) in the TG mice. Moreover, an index of LV myocardial stiffness, Young’s modulus *E_H_*, was also increased in the TG mice (*Figure 3D*). LV mRNA and protein levels of Collagen I, TGF-β1 and CTGF were also increased in the TG mice (*Figure 4A* through *F*). The LV IL-16 mRNA level was positively correlated with both the extent of LV fibrosis and *E_H_* in the TG mice (*Figure 4G and H*).

**Table 5:** Comparison of anatomical and functional characteristics of the TG and Non-TG mice.

	Non-TG (*n* = 8)	TG (*n* = 7)
Body weight, g	36.8±1.2	37.5±1.2
Systolic blood pressure, mmHg	93±2	97±2
Heart rate, bpm	633±22	625±13
LV weight/body weight, mg/g	3.40±0.17	2.95±0.11
Atrial weight/body weight, mg/g	0.25±0.01	0.39±0.05*
Lung weight/body weight, mg/g	4.12±0.25	4.27±0.13
**Echocardiography**		
LV end-diastolic dimension, mm	3.76±0.10	3.84±0.11
PWd, mm	0.98±0.03	0.95±0.04
Fractional shortening, %	41±2	43±1

Data are mean ± SEM. LV indicates left ventricular; and PWd, LV posterior wall thickness at end-diastole.

* *P*<0.05 vs. Non-TG mice.

### Macrophages are Involved in Cardiac Fibrosis Caused by Enhanced Cardiac Expression of IL-16

**Figure 5:**
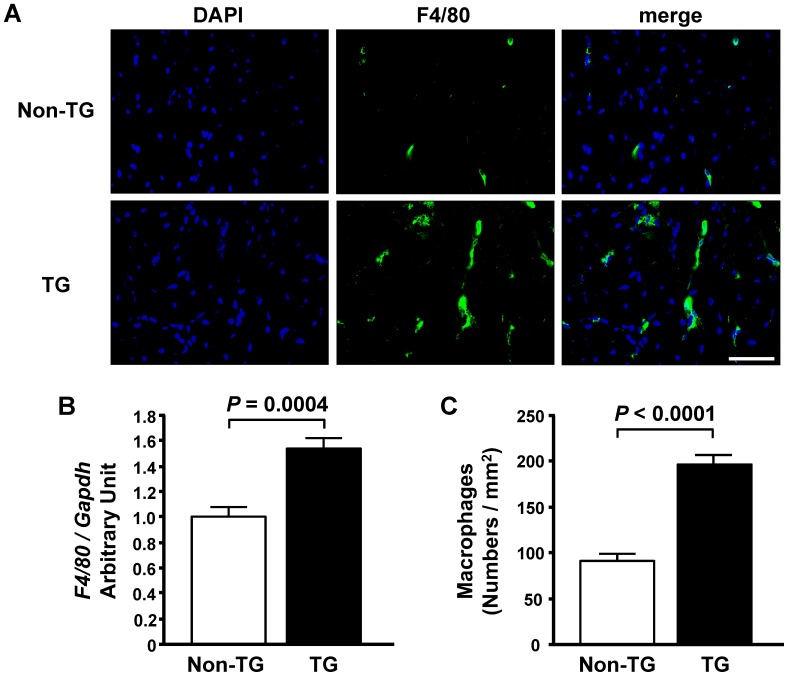
Enhanced cardiac expression of interleukin-16 induces cardiac macrophage infiltration in mice. **A**, Representative photomicrographs of immunofluorescence staining of the left ventricle for F4/80 in non-transgenic (Non-TG) and transgenic (TG) mice. Bar = 50 µm. **B**, Left ventricular mRNA levels of F4/80 in Non-TG and TG mice. **C**, Quantitative analysis of macrophage infiltration into left ventricular myocardium in Non-TG and TG mice. *n* = 6 per group.

**Figure 6:**
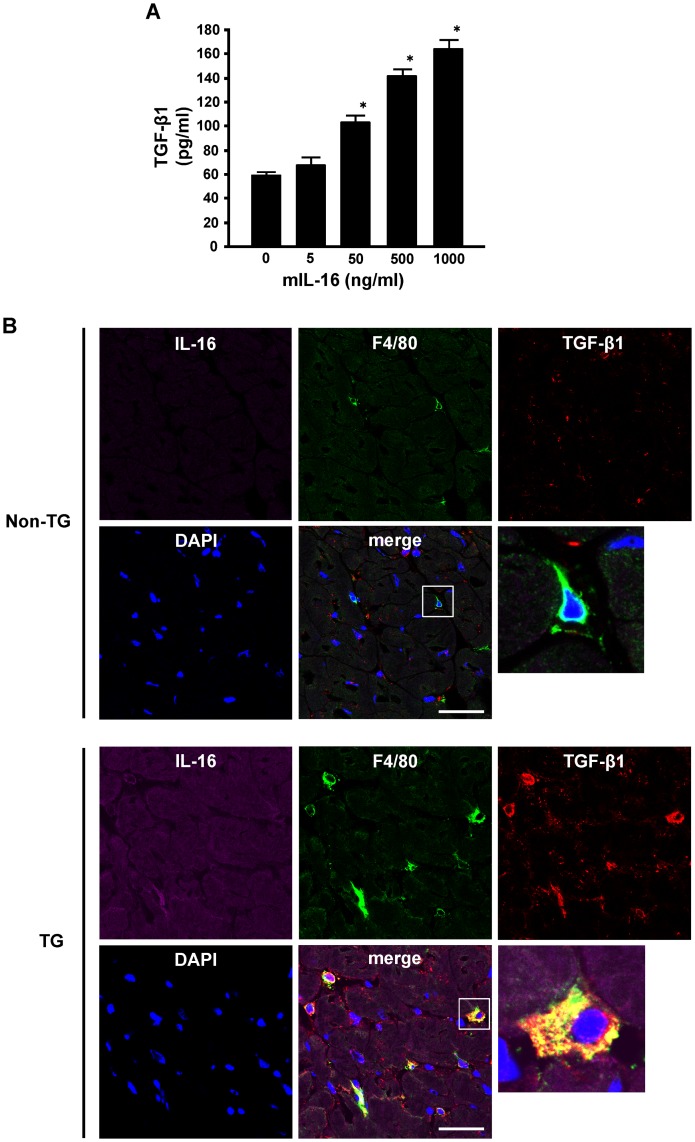
Effect of interleukin-16 (IL-16) on transforming growth factor-beta 1 (TGF-β1) production by macrophages. **A**, Production of TGF-β1 by macrophages treated with murine recombinant IL-16 (mIL-16). Mouse peritoneal macrophages (2×10^6^ cells/well) were treated with the indicated concentration of mIL-16 for 24 hours. The supernatants of macrophage cultures were collected and TGF-β1 was measured as described in the Methods. *n* = 5 for each group. *P<0.05 vs. 0 ng/ml group. **B**, Representative photomicrographs of confocal immunofluorescence staining of the left ventricle in non-transgenic (Non-TG) and transgenic (TG) mice for IL16 (violet), F4/80 (green), and TGF-β1 (red).

Inflammatory cells, especially infiltrating monocytes and macrophages in the heart, have been suggested to have a crucial role in cardiac fibrosis [17], [37], [38], whereas IL-16 has been reported to be able to chemoattract monocytes [39]. Therefore, we examined macrophage infiltration into LV myocardium of TG mice and assessed the direct effect of IL-16 on cultured macrophages. Macrophages were significantly increased in the left ventricle of TG mice (*Figure 5A* through *C*). In addition, when stimulated with recombinant murine IL-16, mouse peritoneal macrophages released TGF-β1 in a dose-dependent manner (*Figure 6A*). Confocal immunofluorescence microscopy revealed that many F4/80-positive macrophages colocalized with TGF-β1 in the left ventricle of TG mice compared with Non-TG mice (Figure 6B). These data suggested that IL-16 might be at least one of the cytokines playing a central role in the promotion of cardiac fibrosis through the release of TGF-β1 from the infiltrating and resident macrophages in TG mice.

### Neutralization of IL-16 Ameliorates the Development of Cardiac Fibrosis

**Figure 7:**
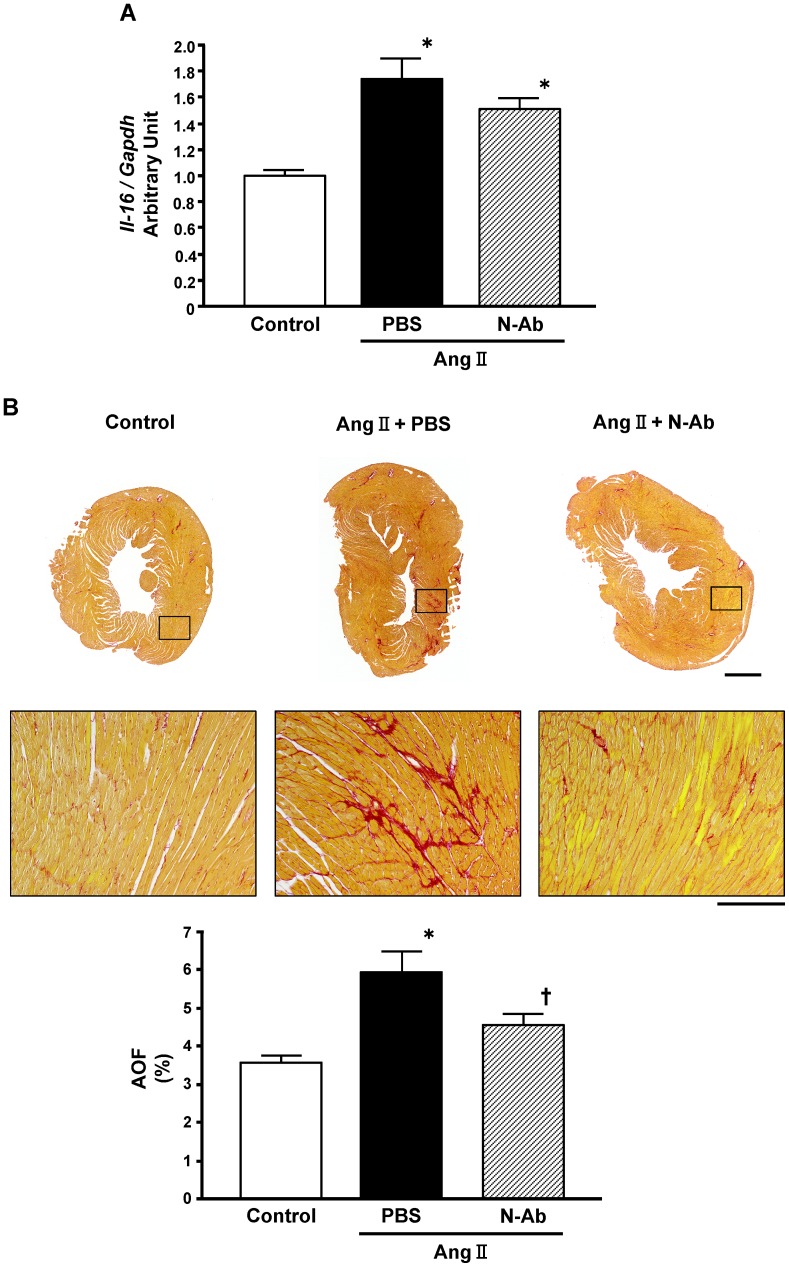
Neutralization of interleukin-16 (IL-16) ameliorates the development of cardiac fibrosis. **A**, mRNA level of IL-16 in the left ventricle of control mice and mice injected intraperitoneally with phosphate buffered saline (PBS) or anti-IL-16 neutralizing antibody (N-Ab) during angiotensin II (Ang II) infusion for 14 days. *P<0.05 vs. control group. **B**, Representative photomicrograph of Sirius Red-stained heart sections and the percent area of fibrosis (AOF) in control mice and mice injected intraperitoneally with PBS or N-Ab during Ang II infusion for 28 days. Bar: Upper panel = 1 mm; Lower panel = 200 µm. *P<0.05 vs. control group. ^†^ *P*<0.05 vs. mice with PBS treatment during Ang II infusion.

Chronic infusion of Ang II induces hypertension and cardiac fibrosis, and has been used as a model to explore the mechanisms underlying the fibrotic process in the heart. Using this model, we examined the effect of IL-16 neutralization on cardiac fibrosis. After 14 days of Ang II infusion, IL-16 mRNA was significantly upregulated in the LV myocardium (*Figure 7A*). Neutralization of IL-16 had no effect on IL-16 mRNA levels, suggesting that neutralization of IL-16 neither promotes nor suppresses IL-16 expression in cardiac tissue (*Figure 7A*). Histopathology of the heart from the mice infused with Ang II for 28 days showed marked cardiac fibrosis, which was significantly attenuated by anti-IL-16 neutralizing antibody therapy without any effect on systemic blood pressure (*Table 6* and *Figure 7B*). Atrial weight and lung weight were also decreased by IL-16 neutralization, suggesting that IL-16 neutralization might have inhibited the increase in LV filling pressure and pulmonary congestion (*Table 6*).

**Table 6:** Comparison of control mice and angiotensin II-infused mice treated with PBS or anti-IL-16 neutralizing antibody.

	Control (*n* = 12)	Ang II+PBS (*n* = 12)	Ang II+N-Ab (*n* = 11)
Body weight, g	27.2±0.3	25.2±0.3*	25.1±0.3*
Systolic blood pressure, mmHg	102±1	152±5*	159±5*
Heart rate, bpm	679±7	678±13	665±9
LV weight/body weight, mg/g	3.49±0.06	4.87±0.14*	4.67±0.19*
Atrial weight/body weight, mg/g	0.25±0.01	0.38±0.02*	0.33±0.01* †
Lung weight/body weight, mg/g	4.86±0.05	5.61±0.09*	5.38±0.04* †
**Echocardiography**			
LV end-diastolic dimension, mm	3.75±0.06	3.26±0.04*	3.20±0.06*
PWd, mm	0.82±0.01	1.10±0.02*	1.10±0.04*
Fractional shortening, %	44±1	42±1	45±2

Data are mean ± SEM. Ang indicates angiotensin; LV, left ventricular; N-Ab, anti-IL-16 neutralizing antibody; PBS, phosphate buffered saline; and PWd, LV posterior wall thickness at end-diastole.

* *P*<0.05 vs. control group,

† *P*<0.05 vs. Ang II+PBS group.

## Discussion

Serum IL-16 levels were elevated in both HFpEF patients and the HFpEF rat model, and closely associated with parameters of LV diastolic dysfunction and LV stiffening. Cardiac expression of IL-16 was augmented in the HFpEF rats. Cardiac-enhanced expression of IL-16 in mice promoted LV fibrosis and LV myocardial stiffening in proportion with IL-16 expression levels. Neutralization of IL-16 ameliorated cardiac fibrosis in the mouse model of Ang II-induced hypertension.

IL-16 was first described as a T-cell chemoattractant generated from human peripheral blood mononuclear cells [40]. Since then, IL-16 has been shown to be associated with various inflammatory, allergic or infectious diseases [19]–[21], but the role of IL-16 in the pathophysiology of heart failure has not been previously reported. In the present study, we showed the first evidence that IL-16 mediates cardiac inflammation leading to increased cardiac fibrosis and LV stiffness. Moreover, serum IL-16 levels were elevated in HFpEF patients compared with the control group but not in HFrEF patients, suggesting that IL-16 might be a specific inflammatory mediator associated with the development of HFpEF.

In the LV myocardium of IL-16 TG mice, we found a significant increase in macrophages, which has been previously suggested to be involved in the development of cardiac fibrosis [17], [37], [38]. However, we did not find an increase in T-cells (data not shown). This was unexpected since CD4 has been reported to be the primary cell surface receptor for IL-16, and increased infiltration of CD4^+^ T-cells has been reported at the site of IL-16-mediated inflammation [20]. IL-16 has also been reported to use receptors other than CD4 [41], but we could not identify the receptor responsible for IL-16-mediated chemoattraction of macrophages into the heart. Although it is likely that IL-16 secreted in the heart causes infiltration of macrophages into the myocardium, further investigation is needed to clarify the difference in IL-16-induced chemoattraction of cells between the heart and other organs.

IL-16 has been shown to induce the production of several cytokines and chemokines from monocytes/macrophages [42], but this is the first report to demonstrate that IL-16 promotes the release of the profibrotic cytokine TGF-β1 from macrophages. Therefore, our results suggest that IL-16 might promote cardiac fibrosis in HFpEF through chemoattraction of macrophages into the myocardium and subsequent stimulation of the release of TGF-β1 from macrophages. Blocking the effect of IL-16 might inhibit this pathway, resulting in the amelioration of cardiac fibrosis and reduced LV stiffness.

Recently, we have reported that a decreased DWS, which is calculated from the movement of the epicardial edge of the LV free wall during diastole, reflects LV wall stiffening [23] and predicts a poor outcome in HFpEF patients [26]. In this study, we found a correlation between serum IL-16 levels and DWS in human subjects, suggesting an association between serum IL-16 levels and LV myocardial stiffness. In addition, we found a correlation between serum IL-16 levels and MSC in rats, suggesting a similar association between IL-16 and LV myocardial stiffness. This study suggested that the elevation of circulating levels of IL-16 in HFpEF could not be explained by the overexpression of IL-16 in the heart, and failed to clarify the source for the elevation; however, measurement of circulating levels of IL-16 might be useful as a surrogate biomarker for estimating the extent of LV myocardial stiffening in HFpEF patients and for risk stratification of patients with HFpEF. Future clinical studies with a larger number of subjects are required to investigate this hypothesis.

In summary, our present work provides the first evidence that IL-16 mediates cardiac inflammation leading to increased cardiac fibrosis and LV stiffness in HFpEF. Blockade of IL-16 may be a possible therapeutic strategy to treat HFpEF.
